# Nasal carriage rate of methicillin resistant *Staphylococcus aureus* among Dessie Referral Hospital Health Care Workers; Dessie, Northeast Ethiopia

**DOI:** 10.1186/2047-2994-2-25

**Published:** 2013-10-02

**Authors:** Agumas Shibabaw, Tamrat Abebe, Adane Mihret

**Affiliations:** 1 Department of Microbiology and Immunology, Faculty of Medicine, Wollo University, Dessie, Ethiopia; 2 Department of Microbiology, Immunology and Parasitology, School of Medicine, Addis Ababa University, Addis Ababa, Ethiopia; 3 Armauer Hansen Research Institute, Addis Ababa, Ethiopia

**Keywords:** MRSA, Healthcare workers, Nasal carriage, *S. aureus*

## Abstract

**Background:**

*Staphylococcus aureus* is a common cause of community and hospital acquired infections. One of the important sources of staphylococci for nosocomial infection is nasal carriage among hospital personnel. Emergence of drug resistant strains especially methicillin resistant S*taphylococcus aureus* is a serious problem in hospital environments. The aim of this study was to determine the nasal carriage rate of methicillin resistant *Staphylococcus aureus* among Dessie Referral Hospital healthcare-workers in Ethiopia.

**Methods:**

A cross sectional study was conducted on a total of 118 healthcare workers. Nasal swabs were collected and cultured on Mannitol Salt Agar. Slide coagulase test was performed. An oxacillin susceptibility test was carried out on Muller Hinton agar using modified Kirby-Bauer disc diffusion method.

**Results:**

Of the 118 healthcare workers, 34 (28.8%) carried *S. aureus* of which 15 were methicillin resistant. Therefore, 12.7% of all HCWs were identified as MRSA carriers. The rate of methicillin resistance among all *S. aureus* isolates was 44.1% (15/34). MRSA carriage was particularly high among nurses (21.2%). The highest rate of MRSA carriers (57.1%) were workers of surgical wards.

**Conclusions:**

The high rate of nasal MRSA carriage among healthcare workers found in this study indicates the need for adjusted infection control measures to prevent MRSA transmission in our healthcare setting.

## Introduction

*Staphylococcus aureus* is a major cause of community and healthcare infections, and methicillin-resistant *S. aureus* (MRSA) is currently the most commonly identified antibiotic-resistant pathogen in many parts of the world. Treatment of infection caused by *S. aureus* has become more problematic since the occurrence of methicillin resistance, as MRSA strains are resistant to all β-lactam antibiotics thereby significantly limiting the treatment options. Concerning Africa, several countries reported MRSA as a problem [1-4], but there is still a lack of good epidemiologic data.

With a few exceptions, the incidence of nosocomial infection caused by MRSA continues to increase worldwide [5]. Infections caused by MRSA strains are associated with longer hospital stay, prolonged antibiotic administration, and higher cost than infections caused by methicillin-susceptible *Staphylococcus aureus* strains. Next to colonized patients and contaminated environmental surfaces, colonized healthcare workers (HCWs) can act as a reservoir for the spread of MRSA to patients and other HCWs [6].

Identification of patients and healthcare workers (in outbreak settings) colonized with MRSA, combined with hand hygiene and other precautions have been shown to be effective in reducing the transmission and controlling the spread of MRSA. In the current study we conducted a cross sectional study to determine the nasal carriage rate of MRSA among HCWs of Dessie Referral Hospital in Ethiopia.

## Materials and methods

The study was carried out from November 2010 to March 2011 in Dessie Regional Health Research laboratory. A cross sectional study was conducted on a total of 118 Dessie referral hospital HCWs. The study participants were selected proportionally according to the number of HCWs in each ward/department. Out of 205, 118 HCWs were enrolled in the study.

### Nasal swab collection

Sterile cotton swabs were used for sample collection. The sample was obtained by rotating the swabs gently for five times on both nares of the study participants so that the tip is entirely at the nasal osteum level and it was transported to the laboratory using Stuart transport media.

### Culture and identification

Swabs were inoculated on to Mannitol Salt Agar (MSA) (OXOID, UK) within one hour of collection and were incubated at 37°C for 24–48 hrs. After the plates were left at room temperature, coagulase test was performed using slide test method [7]. Those colonies that were mannitol fermenter (golden or cream colour on MSA) and tested coagulase positive were taken as *S. aureus.*

### Antimicrobial susceptibility testing

Laboratory antimicrobial susceptibility testing was performed using modified Kirby- Bauer disc diffusion method which is recommended by the Clinical and Laboratory Standards Institute (CLSI) [7]. Colonies confirmed to be *S. aureus* were suspended in tryptone broth until matching with a standard turbidity (0.5 MacFarland). The suspension was used to inoculate a Mueller-Hinton agar (MHA) (OXIOD, UK). An oxacillin disk (1 μg) (OXIOD, UK) was placed on the plates and the plates were incubated for 24 hours at 35°C. Colonies with an inhibition zone of under 11 mm were read as “methicillin” resistant. In this study *Staphylococcus aureus* ATCC 25923 was used for the control [7].

### Statistical analysis

The findings were statistically analyzed using descriptive statistics, Chi-square test (X^2^) and p-value (p < 0.05, statistical significant). The risk factor analysis of MRSA colonization was performed using SPSS (16^th^ version) package.

### Ethical approval

The study was approved by the institutional review board of Medical School of Addis Ababa University and all participants provided their written informed consent before participating in the study.

## Results

### Socio-demographic characteristics

A total of 118 health care workers were involved. The age ranged between 20 and 55 years (mean = 30.4±8.8); 57 (48.3%) were males and 61 (51.7%) were females. The mean number of years in service was 6.1±8.5.

### Rate of isolation

Overall, 28.8% of the HCWs were *S. aureus* carriers (MSSA and MRSA), ranging from 20% (pharmacists) to 37.5% (doctors). A total of 15 MRSA were isolated from 118 participants, giving an overall positivity rate of 12.7%. Among the 57 males screened, 21 (36.8%) and 10 (17.5%) were positive for *S. aureus* and MRSA respectively, compared to 13 (21.3%) and 5 (8.2%) of the 61 females.

### Professional distribution of isolates

The distribution of *S aureus* and MRSA carriage across profession is presented in Table 1. MRSA carriage was particularly high among nurses (21.2%), doctors (12.5%) and technicians (12.5%). Midwives, pharmacists and radiologists (together 17 HCWs) showed no MRSA carriage.

**Table 1:** **Distribution of *S. aureus *and MRSA carriage among the different professions**

**Profession/**	**No**	***S. aureus ***	**MRSA (n=15)**	
**occupation**	**sampled**	**(n=34)**		
		**No (%)**	**No (%)**	**Total % (n=118)**
Doctors	8	3 (37.5)	1 (12.5)	0.85
Nurses	52	18 (34.6)	11 (21.2)	9.3
Midwives	9	2 (22.2)	0 (0.0)	0.0
Technicians	8	2 (25.0)	1 (12.5)	0.85
Nurse Students	33	8 (24.2)	2 (6.1)	1.7
Pharmacist	5	1 (20.0)	0 (0.0)	0.0
Radiologist	3	0 (0.0)	0 (0.0)	0.0

**Figure 1:**
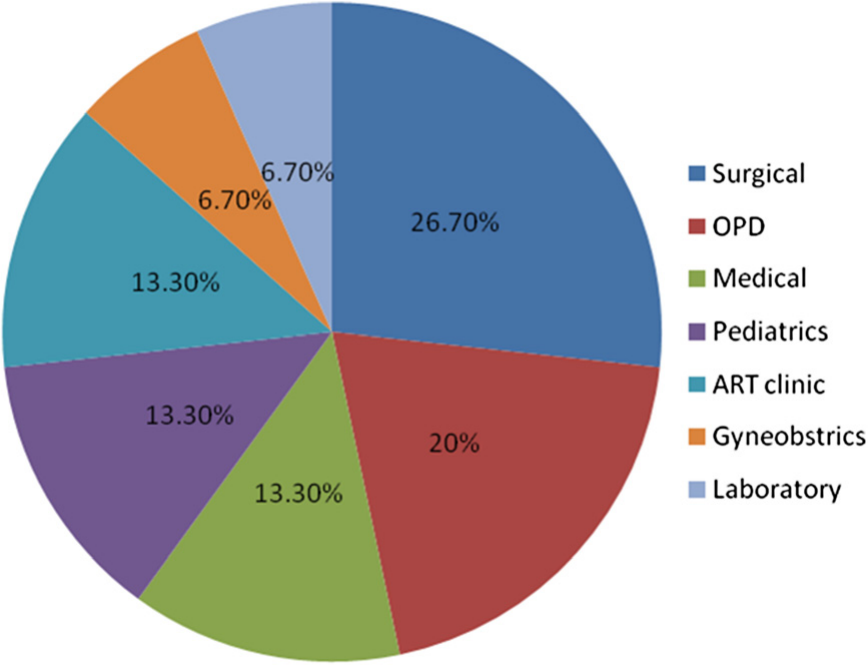
Percentage of MRSA isolates among the different wards/departments.

Based on their areas of work, the highest rate of *S. aureus* carriers (35.7%) and MRSA carriers (57.1%) were among HCWs in pediatrics and surgery, respectively (Table 2). Among the different specialties with patient contact, gynaecology and obstetrics had the lowest rate of MRSA carriage (Figure 1).

**Table 2:** **Percentages of *S. aureus (SA) *and MRSA isolates in relationship with hospital’s wards/departments**

**Ward/**	**No of samples**	**MRSA (%)**	**SA (%)**
**department**	**(n=118)**	**(n=15)**	**(n=34)**
Medical	18	2 (50)	4 (22.2)
Surgical	20	4 (57.1)	7 (35.0)
Pediatrics	14	2 (40)	5 (35.7)
Gyn-Obs	13	1 (25)	4 (30.8)
Laboratory	8	1 (50)	2 (25.0)
OPD	22	3 (50)	6 (27.3)
Pharmacy	5	0 (0.0)	1 (20.0)
X-ray	3	0 (0.0)	0 (0.0)
ART clinic	12	2 (50)	4 (33.3)
Emergency	3	0 (0.0)	1 (33.3)

### Risk factors associated for MRSA colonization

None of the risk factors assessed were significantly associated with the carriage rate of MRSA (Table 3). Highest positive rate was observed in the age group 20 to 29 years (5.9%), followed by 30 to 39 years (5.1%) and 40 to 49 years (1.7%) (p > 0.05). The MRSA prevalence rate for male HCWs 8.5% was twice time that of females HCWs (4.2%), but not statistically different (p > 0.05).

**Table 3:** Risk factors associated with MRSA colonization during the study period

**Associated factor**		**MRSA**	**p-**
		**carriers**	**value**
		**No. (%)**	
Age group	20-29	7 (5.9)	0.13
	30-39	6 (5.1)	
	40-49	2 (1.7)	
	50-59	0 (0)	
Sex	Female	5 (4.2)	0.128
	Male	10 (8.5)	
Ward/ department	Medical	2 (1.7)	0.960
	Surgical	4 (3.4)	
	Pediatrics	2 (1.7)	
	Gyn-Obs	1 (0.8)	
	Laboratory	1 (0.8)	
	OPD	3 (2.5)	
	Pharmacy	0 (0)	
	X-ray	0 (0)	
	ART-clinic	2 (1.7)	
	Emergency	0 (0)	
Profession/ occupation	Doctors	1 (0.8)	0.309
	Nurses	11 (9.3)	
	Midwives	0 (0)	
	Technicians	1 (0.8)	
	Students	2 (1.7)	
	Pharmacist	0 (0)	
	Radiologist	0 (0)	
No. of years in service or practice	≤ 5	9 (7.6)	2.66
	> 5	6 (5.1)	
Hand washing habit	Always	4 (3.4)	0.184
	Sometimes	11(9.3)	
	Rarely	0 (0)	
Habit of cleaning contaminated surfaces/tables	Always	6 (5.1)	0.079
	Sometimes	9 (7.6)	
	Rarely	0 (0)	

## Discussion

In the present study, the nasal carriage of rate staphylococci varied among the different health professionals. The overall nasal carriage rate of *S. aureus* and MRSA was 28.8% and 12.7% respectively. The nasal carriage rate of *S. aureus* in the present study is lower than the study conducted in Gaborone hospital, Botswana (35.8%) [4] and Valdivia Hospital, Chile (34.9%) [8], and comparable to other studies conducted in Chile (27.5%) [9]. In Nepal (25%) [10] and 18.3% in Nairobi hospital, Kenya [3]. All differences between countries and hospitals may be explained by microbiological methods (from sampling technique to culture media), local infection control standards, and the local prevalence of MRSA.

From 34 *Staphylococcus aureus* isolates, 15 were oxacillin/ methicillin resistance with positivity rate of 44.1%. This is far higher than 19% in Libyan hospitals [2], 11.38% in Tehran health care workers [11] and 5.5% in Ghaemshahr hospital personnel [12]. Among MRSA carriers, the highest rate was observed in the age group 20 to 29 years (5.9%) compared with other age groups (p > 0.05). Moreover, the carrier rate was higher in health care workers (7.6%) with < 5 years service or practice experience (p > 0.05). This higher prevalence among the younger and less practiced HCWs could be doe to their lack of knowledge with regard to infection control policies and their missing experience in taking care of these patients.

The MRSA carriage recorded in this study is higher than results obtained from surgical staff members or from environmental contamination of Tikur Anbessa Specialized Hospital in Ethiopia (27.6%) [13]. Even lower rates 38.7% and 40%, were reported from Abidjan [14] and Nepal [10], respectively. A study conducted in Nigeria among health care workers indicated a higher rate (52.5%), than the current study [15].

Obviously, all the above mentioned studies are not fully comparable, since the use of HCWs from specific specialties, and the differences in the study design such as sample size and method of MRSA identification might account for the disparity in the carriage rate. In additions, carrier rates might be influenced to poor personal hygiene of study participants, poor sanitation of the hospital and difference in sampling techniques.

In our study, males (17.5%) were two-times more likely to be MRSA carriers than females. Only one other study has found a marginally higher prevalence of MRSA carriage in males, although it was not statistically significant [16]. Whether this is due to better commitment with infection control and hygienic practice of females, or other factors should be looked at in future studies.

MRSA carriage was particularly high among nurses (21.2%), doctors (12.5%) and laboratory technicians (12.5%). In addition to the higher overall rate of MRSA carriage, the Nigerian study [17] showed a different distribution among the professions, with doctors and nurses being equally and highly colonized (65.2% and 64.2%), respectively. The higher MRSA rate among nurses and physicians could possibly be explained by the high frequency of patient contact among these professionals.

## Conclusions

The present study indicates high nasal carriage rate of MRSA (44.1%) among health care workers. The carriage rate was worse among nurses (21.2%) than doctors (12.5%). The highest rate of MRSA carriers were workers of surgical wards (57.1%). The lowest rate of MRSA carriers was in gynecology and obstetrics (25%).

In hospitals, HCWs nasal carriage of MRSA must be regularly screened and give an early warning of the presence of antimicrobial resistant pathogens. Measures be taken to control the spread of MRSA infection should include: laboratory based surveillance, isolation of colonized and infected patients, use of barrier precautions and basic infection control measures, and screening and treatment of MRSA-positive HCWs.

## Abbreviations

MRSA: Methicillin-resistant *Staphylococcus aureus*; SA: *Staphylococcus aureus*; S. aureus: *Staphylococcus aureus*; HCWs: Health care workers.

## Competing interests

The authors declare that they have no any competing interests.

## Authors’ contributions

AS designed the study, gathered and interpreted data, performed statistical analysis, and drafted the manuscript. TA and AM conceived the study, participated in coordination and interpretation of data, and helped to draft the manuscript. All authors read and approved the final manuscript.

## Authors’ information

We are doing research on infectious diseases including *Staphylococcus.*
